# Can the prophylactic quadrivalent HPV vaccine be used as a therapeutic agent in women with CIN? A randomized trial

**DOI:** 10.1186/s12889-020-8371-z

**Published:** 2020-02-27

**Authors:** Mojgan Karimi-Zarchi, Leila Allahqoli, Ameneh Nehmati, Abolfazl Mehdizadeh Kashi, Shokouh Taghipour-Zahir, Ibrahim Alkatout

**Affiliations:** 1 Endometriosis Research Center, Iran University of Medical Sciences (IUMS), Tehran, Iran; 2 Shahid Sadoughi University of Medical Science, Yazd, Iran; 3 Islamic Azad University, Yazd Branch, Yazd, Iran; 4 Department of Obstetrics and Gynecology, University Hospitals Schleswig-Holstein, Campus Kiel, Kiel, Germany

**Keywords:** HPV, Vaccine, Pap smear, Cervical cancer, Secondary prevention

## Abstract

**Background:**

Human papillomavirus (HPV) is one of the most significant risk factors for cervical cancer. The HPV vaccine has a very significant impact on the incidence of cervical cancer. The present study aimed to investigate the impact of prophylactic quadrivalent HPV vaccine in the treatment of women with cervical intraepithelial neoplasia (CIN 1–3).

**Methods:**

This randomized controlled trial was conducted in the Shahid Sadoughi University of Medical Sciences (SSUMS), Yazd, Iran, from October 2011 to November 2015 in women with histologically confirmed residual/recurrent CIN 1 or high-grade CIN (CIN 2–3). Eligible women were assigned randomly to an intervention and a control group. Women in the intervention group were given HPV vaccinations while those in the control group were not. Participants were followed up for 24 months. Primary and secondary outcomes, and adverse effects of the treatment in the two groups were compared using Student’s *t* test, the chi-square test, or Fisher’s exact test. *P* values < 0.05 or less were considered statistically significant.

**Results:**

Three-hundred and twelve women were randomized to the two groups; the data of 138 in the intervention group and 104 in the control group were analyzed. The mean age of the women was 32.59 ± 4.85 years. Differences in age, marital status, and grades of CIN weren’t significant between the two groups. At the end of the two-year follow-up period, the number of women with CIN 2–3 in the intervention and control groups was reduced by 75% (from 93 to 23) versus 40% (from 69 to 41). The efficacy of the HPV vaccine in women with CIN 1–3 was 58.7% (*p* = 0.018). No serious adverse effects related to the vaccines were reported.

**Conclusions:**

The prophylactic quadrivalent HPV vaccine after treatment may have a therapeutic effect in women with residual/recurrent CIN 1 or high-grade CIN (CIN 2–3).

**Trial registration:**

Iranian Registry of Clinical Trials, IRCT20190603043801N1. Registered 24 July 2019 – Retrospectively registered, http://www.irct.ir/user/trial/40017/view

## Background

Cervical cancer is one of the most common cancers in women throughout the world [1–3]. More than 500,000 new cases occur every year and cervical cancer accounts for more than 250,000 deaths per year [1, 2]. In Iran, the mean age-standardized mortality rates (ASMR) for cervical cancer were reported as 1.04 per 100,000 [3]. The human papillomavirus (HPV) transmitted by sexual contact was found to be one of the risk factors for cervical, breast [4, 5], anal, and oropharyngeal cancer [6, 7]. Permanent high-risk HPV infections are considered as a major cause of intraepithelial neoplasia (CIN 1–3) [6, 8, 9], and the first stage in the progression of cervical cancer [10, 11]. Such that the prevalence of HPV infection in cervical cancer patients was more highly than healthy Iranian women (76% vs.7%) [3]. The immune system frequently eradicates CIN, but in some cases, cervical cancer emerges from CIN [12]. CIN is classified as mild (CIN 1), moderate (CIN 2), or severe (CIN 3) dysplasia [13, 14].

Conservative treatment, including the loop electrosurgical excision procedure (LEEP) and cold-knife conization, can effectively eradicate CIN 2–3 [15, 16]. However, the recurrence of CIN after conservative treatment has been reported between 5 and 30% [16]. Once the lesions are detected, the patients must be followed up and treated again as required [17–19].

Recent studies support the therapeutic role of the HPV vaccine [16, 20, 21]. It has been seen that pre-surgery HPV vaccination in women with HPV-related diseases significantly reduces the incidence of CIN 2–3 [16, 22]. But the efficacy of the HPV vaccination in preventing subsequent disease after conservative treatment of CIN 2–3 has not been investigated so far [16] .

A population of more than 25 million Iranian women older than 15 years of age are at risk of cervical cancer due to a lack of knowledge about HPV infection [23] and changing trends in sexual behavior [3, 24, 25]. Furthermore, HPV vaccines have not yet been introduced in Iran. On the other hand, the HPV vaccine is costly and its availability limited in the country, while affluent countries, HPV vaccinations are given to all women aged 9 to 26 years [26]. The efficacy of the HPV vaccination in preventing subsequent diseases after conservative treatment in women with CIN1–3 who missed the vaccination before developing the disease has not been investigated. So, this study was designed to assess the impact of prophylactic quadrivalent HPV vaccine in the treatment of women with cervical intraepithelial neoplasia (CIN 1–3).

## Methods

### Study design and patients

**Fig. 1:**
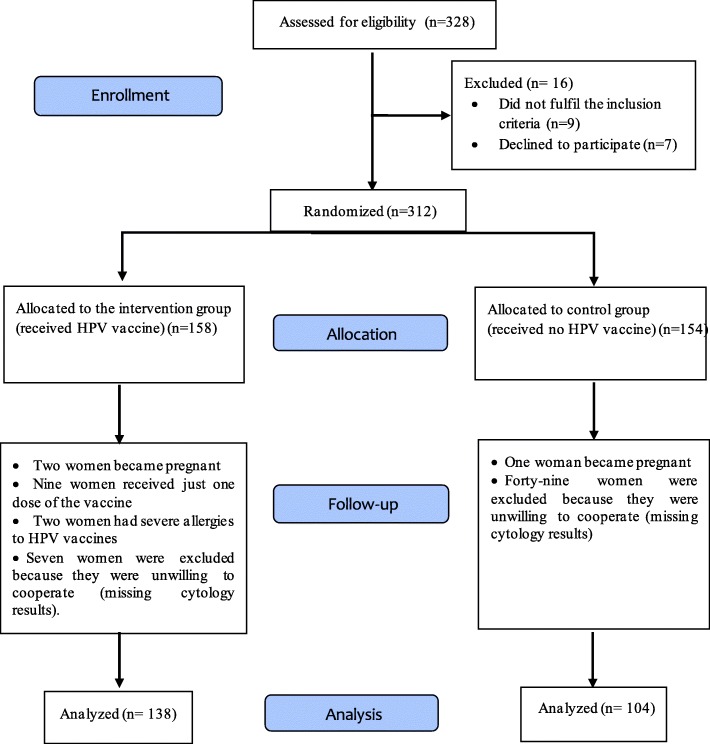
Flowchart of the study

A randomized controlled trial was conducted in gynecological clinics affiliated to the Shahid Sadoughi University of Medical Sciences (SSUMS), Yazd, Iran, from October 2011 to November 2015 to evaluate the effect of HPV vaccine post-surgery in the treatment of women with CIN 1 or high-grade CIN (CIN 2–3). The study sample size was calculated at least 138 women in each group based on the study by the Future II Study Group (Vila), considering p_1_ = proportion of CIN cured by vaccine = 0.183, p_2_ = proportion of CIN cured by placebo 5.6 [27], an alpha error of 0.05 and study power of 80%. In this study, 20% of the sample was assumed as lost to follow up so, the sample size was considered at 150 women in each group. According to the calculated sample size, women who had the following inclusion criteria were enrolled in the study by a convenient sampling method. The inclusion criteria for both groups included women: 1) a) age 21–45 years, b) currently not pregnant, c) no abnormal results on a previous cervical smear test, d) no more than four sexual partners in the course of their lives, e) women with histologically confirmed residual/recurrent CIN 1 or high-grade CIN (CIN 2–3), and f) treated by conservative treatment. Three-hundred and twenty-eight women were assessed for eligibility. Those who did not fulfil the inclusion criteria or declined to participate were excluded (Fig. 1).

### Blinding and intervention

All patients were included in the study after they received sufficient explanation about the study objectives and signed the written informed consent. Three-hundred and twelve women were randomized to the intervention group or the control group through a computer-generated random table of quadruple block numbers (block size of four). One nurse who was not involved in the research prepared the coded envelopes allocated the women into two groups. The main investigator and the gynecologist who assessed the outcomes were blinded to the group allocation. The statistician who analyzed the outcomes was also unaware of the allocations. Women in the intervention group received conservative treatment according to the ASCCP algorithm [28], along with quadrivalent HPV vaccinations (Gardasil). Gardasil (Merck and Co., Inc., Whitehouse Station, NJ, USA) targets HPV types 6, 11, 16, and 18. The vaccine was given as a series of intramuscular injections of 0.5 mL on day 1, month 2, and month 6 [29]. Forty-eight to 72 h after the injection, the patients were checked for skin complications, fever, headache, pain, and inflammation. Women in the control group received treatment according to the ASCCP algorithm [28], without the HPV vaccination.

### Outcomes, measurements, and follow-up

Throughout the 2 years, the patients were managed in accordance with the protocol published earlier [28]. The efficacy of the HPV vaccine in the treatment of CIN 1–3 was defined as a primary outcome. To measure this criterion, the women underwent a gynecological examination, a Pap test, and colposcopy and biopsy on day 1 and at months 7, 12, 18, and 24. At each visit, at least two biopsy specimens were taken at the colposcopy to evaluate the response to treatment despite the normal appearance of the cervix. Biopsy specimens were obtained from various areas using separate instruments. All Pap testing and histologic evaluations were performed in the same laboratory. Pap tests were read using the Bethesda system [30, 31]. The results of visual inspection of the cervix and histological biopsy were recorded on the checklist and each patient’s medical records. A normal cytology and a negative colposcopy were not interpreted as the absence of disease, but a negative histological result of the biopsy was considered to indicate no disease.

Women who became pregnant, received just one dose of the vaccine, had severe allergies to HPV vaccines or were unwilling to cooperate (missing cytology results) were excluded from this study (Fig. 1).

The efficacy of the vaccine is expressed as a proportionate reduction in the disease attack rate (AR). The difference in AR between unvaccinated (ARU) and vaccinated (ARV) persons can be calculated from the relative risk (RR) of disease in the vaccinated group, using the following formula: $\mathrm{VE}=\frac{ARU- ARV}{ARU}\times 100$[32].

As secondary outcomes, we compared the efficacy of two and three doses of the HPV vaccine to treat CIN 1–3 and adverse effects of the HPV vaccine, such as headache, pain, swelling, redness, and skin rash.

### Statistical analysis

All the statistical analysis was performed using the Statistical Package of Social Sciences (SPSS), version 16.0 (SPSS Inc., Chicago, IL., USA). Kolmogorov-Smirnov was used to assess the normality of data distribution. The results for the quantitative variables were reported in mean ± SD format and the ordinal qualitative variables were reported in frequency and percentages. All descriptive data had a normal distribution. Therefore, the student’s t-test was used to compare quantitative parametric variables between the two groups. To compare characteristics of the categorical variables between the two groups, the chi-square test or Fisher’s exact test were used. *P* values < 0.05 or less were considered statistically significant.

## Results

In all 328 women were assessed for eligibility and 312 patients were included in the study. One hundred fifty-eight women were assigned to the intervention group and 154 to the control group. Twenty and 50 women in the intervention and control groups, respectively, were lost during the follow-up period due to pregnancy, lack of cooperation, and allergies to the vaccine. Ultimately, the analysis was done with the data of 138 women in the intervention group and 104 cases in the control group (Fig. 1). The mean age of the women was 32.59 ± 4.85 years. Based on the result, differences in age, marital status, and grades of CIN were statistically significant between the two groups (Table 1). Demographic and clinical characteristics of the two groups are shown in Table 1.

**Table 1:** Demographic and clinical characteristics of the two groups

Variable	Control group (*N* = 104)	HPV vaccine group (*N* = 138)	*P* value
Age, (y)			
Min- Max	22–41	22–42	
Mean ± SD	33.04 ± 4.6	31.7 ± 4.8	0.5*
Marital status; N (%)			
Married	103 (99)	136 (98.5)	
Divorced	1 (1)	2 (1.45)	0.8**
CIN 1; N (%)	35 (33.7)	45 (32.6)	
CIN 2; N (%)	35 (33.7)	50 (36.2)	0.3***
CIN 3; N (%)	34 (32.6)	43 (31.2)	
Two-dose vaccination; N (%)	–	35 (26.4)	–
Three-dose vaccination; N (%)	–	103 (74.6)	

Abbreviations: *Y* year; *N* number; *CIN* Cervical intraepithelial neoplasia; *SD* Standard deviation, *Student’s t test, ** Fisher’s exact test, ***Chi-squared test

At the two-year follow-up, 75.6% of CIN 1 lesions, 78% of CIN 2 lesions, and 72.1% of CIN 3 lesions in women in the intervention group had regressed. The total number of patients with CIN 1, 2, 3, who returned to normal differed significantly in the two groups (*p* = 0.02, 0.03, 0.03). The development of CIN in the two groups after 2 years of follow-up is summarized in Table 2.

**Table 2:** Efficacy of the HPV vaccine in women with CIN after 2 years of follow-up

Variable		Post-injection condition of the lesion after 2 years of follow-up			Efficacy (%)	*P value
		Normal	CIN 1	CIN 2–3		
		N (%)				
CIN 1	Control (*N* = 35)	16 (45.7)	19 (54.3)	–	54.9	0.02
	Two more doses of vaccination (*N* = 45)	34 (75.6)	11 (24.4)	–		
CIN 2	Controls (*N* = 35)	14 (40)	–	21 (60)	63.3	0.01
	Two more doses of vaccination (*N* = 50)	39 (78)	–	11 (22)		
CIN 3	**Controls (*N* = 34)	14 (41.2)	–	20 (58.2)	52.5	0.03
	***Two more doses of vaccination (*N* = 43)	31 (72.1)	–	12 (27.9)		

Abbreviations: *N* Number; *CIN* Cervical intraepithelial neoplasia

* Data were analyzed with Fisher’s exact test

**One woman in the control group actually developed invasive cervical cancer

***All women with CIN 3 received 3 doses of the vaccination

As shown in Table 2, the overall efficacy of the vaccine (two or more vaccinations) in women with CIN 1, 2, 3 was 54.9, 63.3, and 52.5% respectively. The efficacy of two and three doses of HPV vaccine in treating CIN 1 was 38.6 and 63.1%, and their efficacy in treating CIN 2 was 50 and 72.2%, respectively. The difference between the efficacy of different doses of the vaccine for the treatment of residual/recurrent CIN 1 and CIN 2 was statistically significant (*p* = 0.012, *p* = 0.042).

All women with CIN 3 received three doses of the vaccination. The efficacy of the vaccine in women who received three doses was superior to its efficacy in women who received two doses; both of these groups were superior to controls. Data concerning the efficacy of the vaccine in regard to CIN 1–3 are shown in Table 2.

Of 35 patients who received two doses of the vaccine, only one woman (2/9%) experienced a headache. Of 103 patients who were given three doses of the vaccine, 10 (9.7%) reported headache. The difference was not significant on the chi-square test (*p* = 0.191). Women who received two doses of the vaccine reported redness and rash at the injection site in 34 cases (*n* = 35; 97.1%), whereas women who received three doses reported redness and rash in 93 cases (*n* = 103; 90.3%). The difference was not significant (*p* = 0.191) on the chi-square test. No further complications were registered in the study.

## Discussion

To be able to prevent cervical cancer in women aged less than 45 years the quadrivalent HPV vaccine was developed [33]. Recently, the therapeutic role of the HPV vaccine has been claimed in some studies [16, 20, 21]. In the present study, the impact of prophylactic quadrivalent HPV vaccine in the treatment of women with cervical intraepithelial neoplasia (CIN 1–3) was investigated. Based on the result, a 58.7% reduction in the recurrence of CIN 1–3 was reported in women who received two or more doses of the quadrivalent HPV vaccination after the conservative treatment of CIN 1–3. Satisfactory results of therapeutic HPV vaccinations have been also reported in women with CIN 1–3 in previous clinical trials [20, 21].

At the two-year follow-up, 45.7 and 75.6% of CIN 1 lesions regressed in control and intervention groups respectively. Since CIN 1 does not pose a significant risk factor for developing CIN 3, then it is not considered for screening, or treatment [34]. Accordingly, in this study, we just included women with residual/recurrent CIN 1. The overall efficacy of two further doses of the HPV vaccine in the treatment of residual/recurrent CIN 1 was 54.9%. In accordance with these data, the efficacy of the vaccine in the prevention of recurrent CIN 1 lesions has been reported in studies 42.6% [35] and 48.3% [22].

In the present study, at the two-year follow-up, the vaccine reduced the number of women with CIN 2–3 by 75% (93 to 23 women). The overall efficacy of two further doses of the HPV vaccine in the treatment of CIN 2 and CIN 3 was 63.3 and 52.5% respectively. In line with our data, in Joura and co-workers’ study, the efficacy of the vaccination in the reduction of high-grade cervical disease was 64.9% (95% CI 20.1 to 86.3%) [22]. In addition, a prospective nonrandomized study conducted in Korea showed that the post-surgical HPV vaccination has been accompanied by a lower risk of recurrence of CIN 2. So that the recurrence of CIN 2 lesions in HPV vaccinated and non-vaccinated groups was 2.5% (9/360) vs. 7.2% (27/377), (*p* < 0.01) [16].

In the present study, at the two-year follow-up, 22% of CIN 2 lesions in women in intervention group persisted or progressed to CIN 3, and 27.9% of CIN 3 lesions persisted. In Tainio and coworkers’ study 3160 women with CIN 2 lesions were investigated in terms of spontaneous regression, persistence, or progression to CIN 3 or cancer. At the end of the follow-up period, 18% of CIN 2 lesions had progressed, 32% persisted, and 50% had regressed [36].

Since the single dose of the HPV vaccine has not been proven to be effective [37–40], therefore, in the present study women who received just one dose of the vaccine were excluded. The efficacy of two and three doses of the HPV vaccine in the treatment of residual/recurrent CIN 1 was 38.6 and 63.1%, respectively. The efficacy of two and three doses for the treatment of CIN 2 was 50, and 72.2%, respectively. Although the results of some studies suggest that three doses of HPV vaccination is highly more effective than two doses in preventing of occurrence of cervical neoplasia [38], but some studies reported no significant difference between two and three doses [40, 41]. In our study, we noted a big difference in efficacy with three doses of HPV vaccine versus two doses of vaccine. In line with our results, in Basu and coworkers’ study, the efficacy of three and two doses of the vaccine against high-grade lesions in recipients was 46% versus 21% [38]. Since the primary purpose of the present study was not to compare the efficacy of two or three doses of the HPV vaccine, so randomization was not performed on this basis. Therefore, this issue calls for another ethically well-formed longer studies with appropriate design.

In the present study, one woman in the control group actually developed invasive cervical cancer. While, in McCredie and co-workers’ study, invasive cervical cancer occurred in almost 30% of women with untreated CIN 3 developed over a 30-year follow-up period. Women with untreated CIN 3 were at high risk of cervical cancer, while the risk was very low in women who were receiving conservative treatment throughout [42]. The difference observed in our study with McCredie et al. study is that, in our study, all women (intervention or control group) received lesions-related treatment, including LEEP, cold-knife conization, ablation according to protocols [28]. Therefore, the effect of vaccination after the treatment of CIN 1–3 was assessed in women who missed the chance to be vaccinated before developing the disease. In fact, the vaccination is being used to force the immune system to produce antibodies that can block spontaneous HPV infection and reduces the recurrence of the CIN lesions [11, 16, 20, 22]. The results of studies show that immunizing against HPV infection is able to protect patients from precancerous cervical conditions and is very likely to reduce cervical cancer rates in the future [16, 20, 22]. Since the prevention of all types of cervical cancer is not possible with the HPV vaccine, so women still need to go for regular screening even after they have been vaccinated [43].

The side effects of the HPV vaccine in the present study were headaches, redness, and rash at the injection site. The most frequent side effect of the prophylactic quadrivalent HPV vaccine in Goncalves et al. was pain and swelling at the injection site. Other complications included fatigue, headache, fever, and gastrointestinal symptoms in a later stage [44]. Safety outcomes were similar in the various investigated groups [45, 46]. In a follow-up study conducted by Romanowski et al., a serious adverse event was reported by 30 (8%) women in the vaccine group versus 37 (10%) in the placebo group. None was considered to be related or possibly related to the vaccination, and no deaths occurred [45].

Although the present study yielded important data, the limitations are worthy of mention. Due to the high costs of the vaccine, our results are based on the evaluation of a small number of persons. The second limitation was the short duration of follow-up. The third limitation was that we disregarded the women’s HPV status (positive or negative. Finally, the women’s age range and the dosage of the vaccine were not regarded as important factors in the design of the study and their randomization to the control and intervention groups. Studies have shown that the rate of regression of lesions differs in various age groups [47]. Such that the regression rate more highly was reported among women below 30 years of age than (60% vs.11%) [36]. The fact that all women with CIN 3 received three doses of the vaccine might have influenced the results of the present study. Therefore, long-term studies of an appropriate design will be needed to investigate the long-term efficacy of the HPV vaccine in preventing the progression of cervical lesions.

## Conclusions

The present study showed that a prophylactic quadrivalent HPV vaccine actually can be used for therapeutic purposes in women with histologically confirmed residual/recurrent CIN 1 or high-grade cervical intraepithelial neoplasia (CIN 2–3). All women (vaccinated and non-vaccinated) with cervical lesions were followed up for 2 years. The study demonstrated the efficacy of the vaccine in the treatment and resolution of cervical lesions. In fact, nearly a reduction of nearly 60% was noted in the recurrence cervical lesions (CIN 1–3) after two further doses of the vaccine. Our data concur with those of other studies which showed that the efficacy of the vaccine was higher in women who received three doses of the vaccine than in those who received two doses, for both high-grade and low-grade lesions [39, 48].

## Data Availability

The data sets generated and analyzed are available from the corresponding author under reasonable request and with permission from Shahid Sadoughi University of Medical Sciences (SSUMS), Yazd, Iran.

## Abbreviations

ASCCP: American Society for Colposcopy and Cervical Pathology
ASMR: Age-Standardized Mortality Rate
CIN: Cervical Intraepithelial Neoplasia
HPV: Human papillomavirus
LEEP: Loop electrosurgical excision procedure
SPSS: Statistical Package of Social Sciences
VLP: Virus-like particle
